# Magnitude, Trends, and Determinants of Institutional Delivery Among Reproductive Age Women in Kersa Health and Demographic Surveillance System Site, Eastern Ethiopia: A Multilevel Analysis

**DOI:** 10.3389/fgwh.2022.821858

**Published:** 2022-02-28

**Authors:** Temam Beshir Raru, Galana Mamo Ayana, Mohammed Yuya, Bedasa Taye Merga, Mohammed Abdurke Kure, Belay Negash, Abdi Birhanu, Addisu Alemu, Yadeta Dessie, Merga Dheresa

**Affiliations:** ^1^School of Public Health, College of Health and Medical Sciences, Haramaya University, Harar, Ethiopia; ^2^School of Nursing and Midwifery, College of Health and Medical Sciences, Haramaya University, Harar, Ethiopia; ^3^School of Medicine, College of Health and Medical Sciences, Haramaya University, Harar, Ethiopia

**Keywords:** trends, institutional delivery, reproductive aged women, Kersa HDSS, Eastern Ethiopia

## Abstract

**Background:**

Institutional delivery service utilization is a critical and proven intervention for reducing maternal and neonatal mortality. Institutional delivery service utilization can improve maternal health and wellbeing by ensuring safe delivery and reducing problems occurring during childbirth. In Ethiopia, almost all previous researches were cross-sectional studies and most of them were based on small sample sizes and there are no sufficient reports for the trends. Therefore, this study aimed to assess the magnitude, trends, and determinants of institutional delivery using surveillance data from the Kersa Health and Demographic Surveillance System (HDSS), in Eastern Ethiopia from 2015 to 2020.

**Methods:**

The study was conducted among reproductive-aged women selected from the Kersa HDSS site, Eastern Ethiopia for the duration of 2015 to 2020. Data were extracted from the Kersa HDSS database system. After coding and recoding, the data was exported to R software for further analysis. A chi-squared test was used for trends to examine the significance of the change. A multilevel logistic regression model was fitted to identify determinants of institutional delivery. An adjusted odds ratio with a 95% confidence interval (CI) was used to measure the strength of the associations. Statistical significance was declared at a *p*-value < 0.05.

**Results:**

A total of 20,033 reproductive age women were employed for analysis. The overall magnitude of institutional delivery was 45.03% with 95% CI (44.33–45.72). The institutional delivery has shown a decreasing trend over the 6 years' and there is statistical significance for the declining. Semi-urban resident [AOR = 2.33, 95% CI: 1.37–4.48], urban resident [AOR = 7.18, 95% CI: 5.24, 8.71], read and write [AOR = 1.54, 95% CI: 1.18, 2.01], literate [AOR = 1.46, 95% CI: 1.34–1.59], and antenatal care [AOR = 1.73, 95% CI: 1.58–1.88] were significantly associated with institutional delivery.

**Conclusion:**

The magnitude of institutional delivery was relatively low and has shown a decreasing trend. Community-based interventions should be strengthened to reverse the decreasing trend of institutional delivery. Targeted information dissemination and communication should be provided to those mothers who have no formal education and attention should be given to rural residents.

## Introduction

Worldwide, maternal deaths remain an important public health problem, particularly in sub-Saharan Africa. The maternal mortality ratio (MMR) reduced by 44% from 1990 to 2015, with an incidence remaining unacceptably high in developing countries, which accounts for 99% of global maternal deaths (1, 2). Globally, each year, an estimated 303,000 mothers die due to complications related to pregnancy and childbirth. The majority of these maternal deaths occur in low-and middle-income countries (3), with a huge toll of deaths attributed to sub-Saharan Africa and South Asia (3, 4). For instance; every day in 2017, ~810 women died from preventable pregnancy-related complications (5).

Ethiopia is among the countries with a high maternal mortality rate in sub-Saharan Africa. Current evidence from the Ethiopian Demographic Health Survey (EDHS) revealed that maternal deaths represent 25% of all deaths among women aged 15–49 years. In other words, for every 1,000 births in Ethiopia, there are about four maternal deaths due to pregnancy-related complications. In Ethiopia, although the MMR was declining from a huge toll of 676 deaths per 100,000 live births in 2011 to 412 deaths per 100,000 live birth in 2016, still the mortality rate is far from reaching the global target goal (6). Literature has shown that, of the five major causes of maternal mortality, more than 52% of maternal deaths are attributed to three preventable causes such as hemorrhage, sepsis, and hypertension during pregnancy (7, 8). Home delivery is also associated with many of these maternal deaths (9).

Furthermore, in Ethiopia, institutional delivery remains an important public health challenge because only a small proportion of women are delivered at a health facility. In Ethiopia, the magnitude of institutional delivery varies by region and methods of assessment. For instance, different studies that have been conducted in the last 5 years reported low proportions of institutional delivery with a large variation among the nine regions, which ranges from 13.9% in the Pastoral community of Guji Zone, Southern Ethiopia to 78.8% in Bahirdar, Northern Ethiopia (10–15). On the contrary, home delivery also continues to be a significant public health concern at the local and national level (16–18), especially in Pastoralist and semi-Pastoralist communities of Afar and Somali regions (19, 20). On average, three-fourths of Ethiopian women are delivering at home (6, 16).

In addition, researchers have found that numerous factors affect institutional delivery services. For instance, maternal residence, antenatal care visits, exposure to information, educational level, knowledge of mothers on danger signs of pregnancy and institutional delivery services, family size, availability of transport, and planned pregnancy are associated with the enhancement of institutional delivery services utilization (11, 21–24).

Universal access to prenatal care and skilled birth attendants is devised to be the primary strategy of sustainable development goals (SGDs) to end all preventable causes of newborn and maternal deaths by 2030. and to have a global MMR of <70 per 100,000 live births, and to reduce neonatal mortality at least to 12 per 1,000 liver birth in every country by the year 2030 (5, 25).

Ethiopia has also developed various interventions since 2015 to achieve the target of SDGs. For example, in realizing this low institutional delivery, the Ethiopian Federal Ministry of Health (FMOH) set a national goal to increase institutional delivery to 70% by 2025 (Health Sector Transformation Plan II HSTP II 2020/21-2024/25 (2013 EFY - 2017 EFY). In addition, FMOH has also introduced new programs such as health extension programs (HEP) and community health insurance programs (CHIP) to ensure the accessibility of basic healthcare services to the rural community (26, 27). With all these efforts are being implemented, still, the MMR is far from reaching national and global targets. Moreover, although the government has made maximum efforts to improve maternal health service utilization, still there is a significant low utilization of institutional deliveries (6), and a large number of Ethiopian women are delivering at home (28).

In Ethiopia, although institutional delivery has been widely investigated, almost all previous researches were cross-sectional studies and most of them were used small sample sizes, which may not be generalizable to the entire population, and there are no sufficient reports for the trends. Moreover, most of the previous researchers were used logistic regression analysis, in which the assumption of independent observations and equal variance across Kebeles/clusters might be violated. Therefore, this study aimed to investigate the magnitude, trends, and determinants of institutional delivery among women of reproductive age using surveillance data in a community setting in Eastern Ethiopia from 2015 to 2020.

## Methods

### Study Setting, and Design

This study was conducted among women of reproductive age (15–49 years) selected from the Kersa Health Demographic Surveillance System (HDSS) field site of Eastern Ethiopia. Kersa HDSS site is one of the full members of the International Network of Demographic Evaluation of Populations and Their Health (INDEPTH). The site was established in September 2007 in Eastern Hararghe Zone, Kersa district, and then expanded to Harari region Hara town in 2012. Currently, the HDSS is operating among 36 Kebles the lowest administrative units (29). Primarily Kersa HDSS follows an open dynamic cohort study design that longitudinally follows individuals living within a specific geographical boundary.

### Population and Eligibility Criteria

The source population for this study was all mothers aged 15–49 years in Eastern Ethiopia. All mothers aged 15–49 years in Eastern Ethiopia who were in the Kersa HDSS site were the study population. Specific mothers who have no record for the outcome variable were excluded from the study. All mothers aged 15–49 years found in the Kersa HDSS database from 2015 to 2020 who have a measurement for the outcome variable were included. Finally, a total of 20,033 mothers aged 15–49 years were included in this study.

### Data Source and Data Collection Procedure

This study was a secondary data analysis based on Kersa HDSS. The data was obtained from the Kersa HDSS after authorization was granted from Haramaya University, Kersa HDSS office by explaining the goal of our study. Kersa HDSS collects the data by well-trained regular staff through face-to-face interviews using a tablet computer with Open Data Kit (ODK) application. Supervisors were assigned to supervise data collectors in the field. Field supervisors checked data quality before it was sent to the database system. If supervisors found a data quality problem, they sent it back to data collectors for correction. Collected data using a tablet computer in the field was temporarily stored on ODK aggregate. The data manager approved the quality of data and migrated data from temporary storage to the final storage Openhds database system (30). We extracted 6 years (January 2015- December 2020) data from the Kersa HDSS database system for our analysis.

### Measurements

The outcome variable for this study was a place of delivery. This outcome variable was dichotomized and coded as 1 if the women delivered their last birth at a health facility and 0 for those delivered at home.

The extracted independent variables were the age of the mother at first birth, region, religion, place of residence, occupational status, educational status, wealth quantile, ANC visit, gravidity, parity, duration of pregnancy, and current mothers' age.

**Parity:** is the number of children a woman has; if she has just one child she was considered as “prim parous” and if she has more than one child but fewer than five, she was considered as “multipara,” and if she has five/more child she was considered as “grand multipara.”

**Gravidity:** is the number of times that the woman becomes pregnant; if she was pregnant just one times she was categorized as “prim parous” and if she was pregnant more than one but fewer than five times, she was categorized as “multipara,” and if she was pregnant five/more times she was categorized as “grand multipara.”

**Age at first birth:** if the women was <20 years old when she gave birth to her first child, it was labeled as “ <20 years” otherwise labeled as “≥20 years.”

**Ante natal care (ANC):** if the pregnant women visited an ANC unit at least once during her last pregnancy, researchers labeled it as “Yes” otherwise “No”.

**Birth attendant:** is the person who provides basic and emergency care to women and their newborns during last delivery.

#### Maternal Educational Level

Maternal educational level was categorized as “literate” if attended any formal school; if not enrolled in any formal education but can read and write or read, was labeled as “can read and/or write”; if neither able to read nor write was labeled as “neither read nor write.”

#### Wealth Index

Households were given scores based on the number and kinds of consumer goods they own, these scores are derived using principal component analysis. Wealth indexes are calculated from the score of the first component or factor comprising several heavily loaded variables and accounting for the largest variation in the data was categorized into quintiles where each individual falls into three quintiles (1st quintile = poor, 2nd quintile = middle, and 3rd quintile = rich).

### Data Management and Processing

Dependent and independent variables were extracted from the datasets using STATA 14 software. Before analysis, data were cleaned. After coding and recoding of extracted data, the data was exported to R software 3.4.4 for further analysis.

### Statistical Analysis

Descriptive analysis was done to describe the data. Continuous variables were described using mean and standard deviation (Std. dev). The proportion of institutional delivery for each year starting from 2015 and up to 2020 was calculated and the trend at different years was plotted. A chi-squared test for trends was used to examine the significance of change over time.

Kersa HDS data has a hierarchical nature, women within one Kebele maybe like each other more than women in the other Kebele. Due to this, the assumption of independent observations and equal variance across Kebeles/clusters might be violated. Therefore, an advanced statistical model is required to consider the between cluster variability to get a reliable standard error and unbiased estimate.

Furthermore, by considering the dichotomous nature of the outcome variable, multilevel mixed-effect logistic regression was fitted. Model comparison was done based on Akaike and Bayesian Information Criteria (AIC and BIC). A mixed-effect model with the lowest Information Criteria (AIC and BIC) was selected.

The individual and community-level variables that determine institutional delivery were checked independently in the bi-variable multilevel mixed-effect logistic regression model and variables that were statistically significant at *p*-value 0.20 (31, 32) in the bi-variable multilevel mixed-effects logistic regression analysis were considered for the final individual and community level model adjustments. In the multivariable multilevel mixed-effect analysis, variables with a *p*-value ≤ 0.05 were declared as significant determinants of institutional delivery. Intra-class correlation coefficient (ICC) was used to check whether the multilevel model is appropriate and how much of the overall variation in the response is explained by clustering.

Four models were fitted. The first was the null model that did not include exposure variables which were used to verify community variance and provide evidence to assess random effects at the community level. Then Model-I was the multivariable model adjustment for individual-level variables and Model-II was adjusted for community-level factors. In Model-III, the outcome variable was equipped with potential candidate variables from both individual and community-level variables.

The fixed effects (a measure of association) were used to estimate the association between the institutional delivery and explanatory variables and expressed as an odds ratio with a 95% confidence interval. Regarding the measures of variation (random-effects), Community-level variance with standard deviation and intra-cluster correlation coefficient (ICC) was used.

### Model Formulation

The multilevel binary logistic regression model incorporates fixed effects and cluster-specific random effects to account for the within-cluster correlation of clustered data. Therefore, the two-level fixed and random-effect logistic regression model was presented as follow (33):


$$\begin{array}{ll} Logit(Y_{ij}) & =\beta_{0j}+\sum^{\text{​}}\beta X_{i}+\;\Upsilon \;Z_{j}+\varepsilon_{j}\\ \;\beta_{0j} & =\beta_{0}+\mu_{j},\mu_{j}\sim \;N(0,\;\sigma_{\mu }^{2}),\;\\ \;\sigma_{\mu }^{2} & =\text{within}-\text{group}\;\text{variance}\\ \;\varepsilon_{j} & =\varepsilon_{0}+\varepsilon_{j},\;\varepsilon_{j}\sim \;N(0,\;\sigma_{\varepsilon }^{2}),\\ \;\sigma_{\varepsilon }^{2} & =\text{between-groupvariance} \end{array}$$

**Where**: **Y** represents the dependent variable, $X_{i}^{\prime }s$ are level-1 factors (individual level), **β** fixed effect regression coefficient, β_0*j*_ is the cluster random intercept, ε_*j*_ is the residual for each cluster ‘j's, Υ*Z*_*j*_ are level-2 factors (community level) in cluster j and *logit* was a link function of the model.

The proportion of between-group variance ($\sigma_{\varepsilon }^{2}$) to total variance ($\sigma_{\mu }^{2}$ + $\sigma_{\varepsilon }^{2}$) is called the intraclass correlation coefficient (ICC) (34). It is calculated using the formula:


$$\begin{array}{l} ICC\left(\rho \right)=\frac{\sigma_{\varepsilon }^{2}}{\sigma_{\varepsilon }^{2}+\sigma_{\mu }^{2}} \end{array}$$

## Results

### Socio-Demographic Characteristics of Mothers

A total of 20,033 mothers aged 15–49 years were included in the final analysis. The study participants were included from Harar town, Kersa rural, and Kersa semi-urban which accounts for 21.91, 71.34, and 6.74% respectively. The mean (St. dev) age of mothers was 29.32 (7.11) years. Around three-fifths (59.08%), of the women give their 1st birth at the age of fewer than 20 years. The majority of the women 11,404 (56.93%) were unable to read and write and more than three-fourths (76.81%) of them were housewives (Table 1).

**Table 1:** Socio-demographic characteristics of the mothers in Kersa HDSS from 2015 to 2020.

**Variables**	**Frequency (*n* = 20,033)**	**Percentage (%)**
**Mother's current age (years)**		
15–24	5,657	28.24
25–34	9,571	47.78
35–49	4,805	23.99
**Mothers age at 1st birth**		
<20	11,836	59.08
20 and above	8,197	40.92
**Place of residence**		
Urban	4,390	21.91
Rural	14,292	71.34
Semi-urban	1,351	6.74
**Religion**		
Muslim	17,415	86.93
Orthodox	2,327	11.62
Others^+^	291	1.45
**Education**		
Unable to read and write	11,404	56.93
Read and or write	358	1.79
Literate	8,271	41.29
**Occupation**		
House wife	15,388	76.81
Daily laborer	505	2.52
Merchant	802	4.00
Unemployed	2,486	12.41
Paid employer	852	4.25
**Wealth quantile**		
First quintile	6,624	33.07
Second quintile	6,808	33.98
Third quintile	6,601	32.95

+ *Religion including Protestant Christian, Catholic Christian, and Traditional believers*.

### Pregnancy-Related Characteristics of Mothers

Near half 10,141 (50.62%) of the women did not have antenatal care during their last pregnancy. The majority of the women 9,746 (48.65%) have multi number pregnancies. The majority of the women 8,830 (44.08%) was attended/were being cared for by a traditional birth attendant (TBA) during their last delivery (Table 2).

**Table 2:** Pregnancy-related characteristics of the mothers in Kersa HDSS from 2015 to 2020.

**Variables**	**Frequency (*n* = 20,033)**	**Percentage (%)**
**ANC visit**		
Yes	9,892	49.38
No	10,141	50.62
**Parity**		
Primipara	4,050	20.22
Multipara	9,896	49.40
Grand multipara	6,087	30.38
**Gravidity**		
Primipara	3,706	18.50
Multipara	9,746	48.65
Grand multi para	6,581	32.85
**Duration of pregnancy**		
Term	18,777	93.73
Pre term	355	1.77
Post-term	901	4.50
**Birth Attendant**		
TBA^a^	8,830	44.08
Health Professionals	8,793	43.89
Relative_Neighbors	2,410	12.03
**Type of outcome**		
Livebirth	19,636	98.02
Abortion/ miscarriage	189	0.94
Still birth	208	1.04

a *TBA, traditional birth attendant*.

### Magnitude and Trends of Institutional Delivery

**Figure 1:**
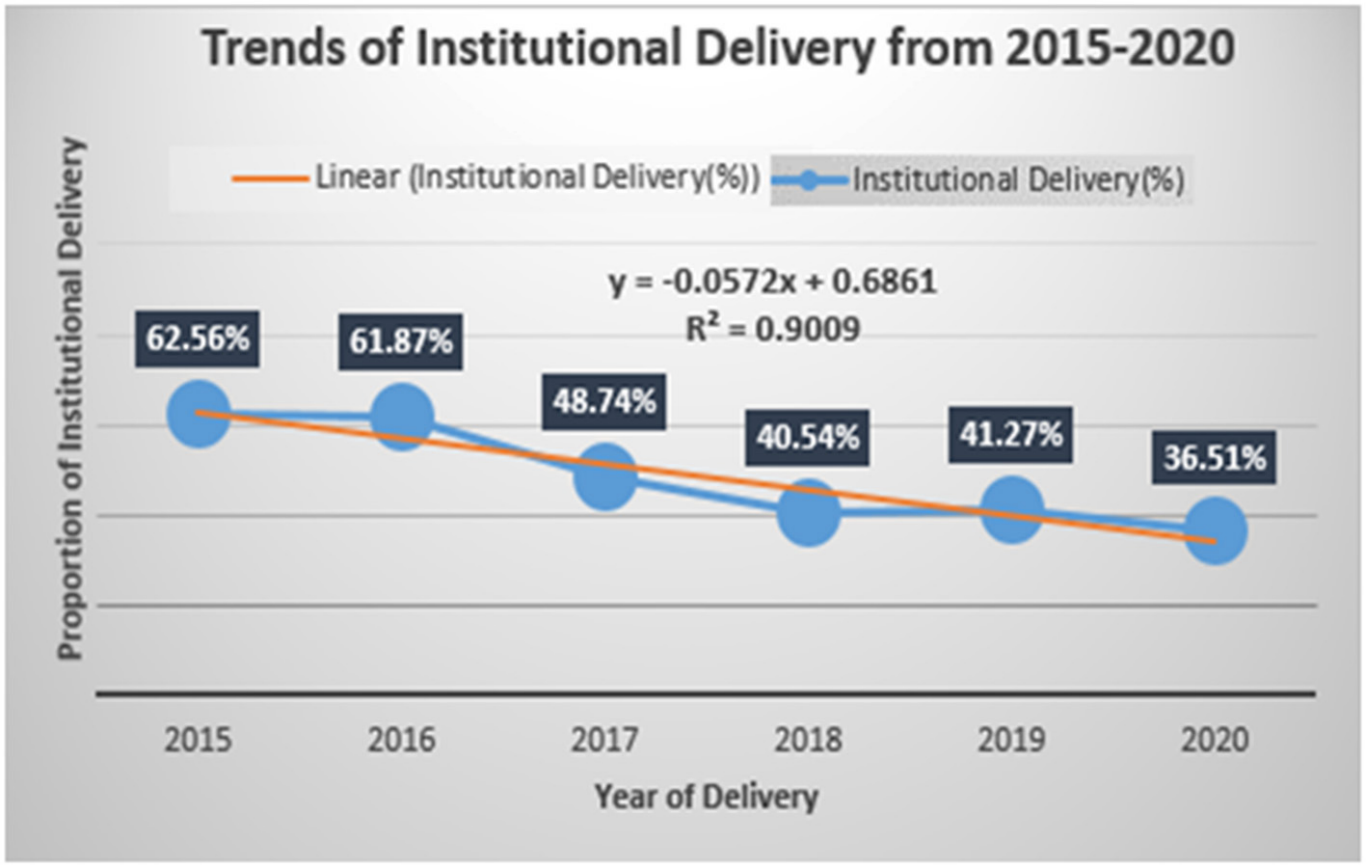
Trends of institutional delivery in Kersa HDSS, 2015–2020.

The overall magnitude of institutional delivery was 45.03% with 95% CI (44.33–45.72). Across the study years, the trend of the institutional delivery varied, the lowest being in 2020 with 36.51% with 95% CI (35.15–37.89) and the highest peak observed in 2015 with 62.56% with 95% CI (60.18–64.88) (Table 3). There was a declining trend in institutional delivery during the reference period in the study area with a slope of 0.0572 on a linear scale (Figure 1), and there is statistical significance for the decline of institutional delivery. Moreover, the study revealed an observed temporal variation in institutional delivery in the study area.

**Table 3:** Magnitude of institutional delivery in Kersa HDSS from 2015 to 2020.

**Place of delivery**	**Frequency (*n* = 20,033)**	**Magnitude (%) and 95% CI**
Home	11,013	54.97% (54.28–55.66)
Health facility	9,020	**45.03% (44.33–45.72)**
**Years of delivery**	**Place of delivery**	
	**Home**	**Health Facility**
2015	37.44% (35.11–39.82)	62.56% (60.18–64.88)
2016	38.13% (36.10–40.21)	61.87% (59.79–63.89)
2017	51.26% (49.45–53.06)	48.74% (46.94–50.55)
2018	59.46% (57.96–60.93)	40.54% (39.07–42.03)
2019	58.73% (57.27–60.18)	41.27% (39.81–42.73)
2020	63.49% (62.11–64.85)	36.51% (35.15–37.89)

*The bold values are indicating the significance association of the predictors*.

### Determinants of Institutional Delivery

In the random effects, the results of the null model revealed that there was statistically significant variability in the odds of institutional delivery with community variance of 6.50 and the ICC in the null model suggested that 66.40% of the total variability in the institutional was ascribed to the differences between communities. In the full model (Model-III: model adjusted for both individual and community-level factors) community variance = 0.49; SE 0.13, remained significant but reduced and 12.98% of the total variance of institutional delivery can be ascribed to the community (Table 4).

**Table 4:** Multivariable multilevel binary logistic regression for institutional delivery among mothers of reproductive age (15–49 years) at Kersa HDSS, 2015–2020.

**Variables**	**Models**			
	**Null model**	**Model-I**	**Model-II**	**Model-III**
		**AOR (95% CI)**	**AOR (95% CI)**	**AOR (95% CI)**
**Residence**				
Rural			**1**	**1**
Semi-urban			1.74 (1.67, 3.46)^*^	2.33 (1.37, 4.48)^*^
Urban			4.56 (2.31, 9.20)^*^	7.18 (5.24, 8.71)^*^
**Residence:Year_Birth**				
Rural:Year_Birth			0.42 (0.35, 0.49)^*^	0.38 (0.32, 0.45)^*^
Semi-urban:Year_Birth			0.51 (0.30, 0.84)^*^	0.72 (0.42, 1.24)
Urban:Year_Birth			2.11 (1.07, 4.11)^*^	2.09 (1.07, 4.09)^*^
**Mother's education**				
Unable to read and write		**1**		**1**
Read and or write		1.54 (1.18, 2.00)^*^		1.54 (1.18, 2.01)^*^
Literate		1.47 (1.35, 1.60)^*^		1.46 (1.34, 1.59)^*^
**Mother's occupation**				
Housewife		**1**		**1**
Daily laborer		8.74 (3.11, 24.59)^*^		1.15 (0.90, 1.47)
Merchant		0.75 (0.29, 1.90)		0.75 (0.29, 1.90)
Unemployed		1.20 (0.80, 1.80)		1.20 (0.80, 1.79)
Paid employer		0.62 (0.20, 1.89)		0.62 (0.20, 1.89)
**Wealth quantile**				
First quantile		**1**		**1**
Second quantile		1.25 (0.93, 1.12)		1.49 (0.79, 2.07)
Third quantile		0.97 (0.88, 1.07)		1.11 (1.02, 1.56)^*^
**Wealth quantile:Year_Birth**				
First quantile:Year_Birth		0.53 (0.41, 0.69)^*^		0.49 (0.39, 0.67)^*^
Second quantile:Year_Birth		0.38 (0.29, 0.50)^*^		0.52 (0.37, 0.67)^*^
Third quantile:Year_Birth		0.48 (0.36, 0.63)^*^		**0.64 (0.47, 0.87)**
**Gravidity^a^**				
Primipara		**1**		**1**
Multipara		1.22 (0.94, 1.58)		1.22 (0.94, 1.57)
Grand multipara		1.23 (0.89, 1.70)		1.23 (0.89, 1.69)
**Parity^b^**				
Primipara		**1**		**1**
Multipara		0.84 (0.65, 1.07)		0.83 (0.65, 1.07)
Grand multipara		0.88 (0.64, 1.21)		0.88 (0.64, 1.21)
**Attended antenatal care**				
No		**1**		**1**
Yes		1.73 (1.59, 1.88)^*^		1.73 (1.58, 1.88)^*^
**Duration of pregnancy**				
Term		**1**		**1**
Preterm		1.13 (0.85, 1.49)		1.13 (0.85, 1.49)
Post-term		0.96 (0.80, 1.15)		0.96 (0.79, 1.15)
**Random effects**				
Community variance	6.50 (1.56)	5.18 (1.26)	0.59 (0.54)	0.49 (0.13)
ICC%	66.40%	61.18%	15.24%	12.98%
**Model comparison**				
AIC	17,250.01	16,856.22	16,942.28	**16,712.82**
BIC	17,265.83	17,188.24	17,092.48	**16,981.59**

a *Gravida (primipara: 1 pregnancy, multipara: 2–4 pregnancy, grand multipara: ≥5 pregnancy)*.

b *Parity (primipara: 1 child, multipara: 2–4 child, grand multipara: ≥5 child)*.

* *p-value < 0.05*.

*COR, Crude odds ratio; AOR, Adjusted odds ratio and 1 stands for reference group. The bold values are indicating the significance association of the predictors*.

In the fixed effects, the model with smaller Akaike Information Criteria (AIC) and Bayesian Information Criteria (BIC) was best fit the data and the interpretation of the fixed effects was based on this model. Model-III was adjusted for both individual and community-level factors and this model fits the data well. In the final model of multi-variable binary logistic regression analysis, variables such as residence, educational status, wealth index, and antenatal care visit were found to be significant determinants of institutional delivery.

The odds of institutional delivery were 2.33 times higher among mothers residing in a semi-urban area [AOR = 2.33, 95% CI: 1.37–4.48], and 7.18 times higher among mothers residing in urban areas [AOR = 7.18, 95% CI: 5.24–8.71] respectively, compared to those who were residing in a rural area. Likewise, the odds of institutional delivery were increased by 54% among mothers who were read and write [AOR = 1.54, 95% CI: 1.18–2.01], and increased by 46% among mothers who were literate [AOR = 1.46, 95% CI: 1.34–1.59], compared to those who were unable to read and write. Regarding the wealth index, the odds of institutional delivery were increased by 11% among mothers who were rich [AOR = 1.11, 95% CI: 1.02–1.56], compared to those who were poor. In addition, the likelihood of institutional delivery was increased by 73% among mothers who receive antenatal care compared to those who do not receive antenatal care service [AOR = 1.73, 95% CI: 1.58–1.88] (Table 4).

Regarding the change in determinants effects over year, place of residence and wealth index were found to be the variables that had significant effects on institutional delivery over the 6 years.

For a 1-year increase in the year of delivery, the likelihood of institutional delivery was decreased by 62% among women residing in rural areas between the year 2015 and 2020 [AOR = 0.38, 95% CI: 0.32–0.45]. Likewise, for a 1-year increase in the year of delivery, the likelihood of institutional delivery was decreased by 51% among poor women during the period 2015 to 2020 [AOR = 0.49, 95% CI: 0.39–0.67] (Table 4).

## Discussion

The overall magnitude of institutional delivery was found to be 45.03%. The institutional delivery has shown a decreasing trend over the 6 years and there is statistical significance for the decline. Factors such as semi-urban residence, urban residence, read and write, literate, rich wealth index, and having antenatal care were the factors that are positively associated with institutional delivery.

The study found the overall magnitude of institutional delivery 45.03% with 95% CI (44.33–45.72). This finding was higher than studies conducted in Afar, Ethiopia (35%) (12), in the Gurage zone, Ethiopia (31%) (35), and lower than studies done in Southwest Ethiopia (76%) (36), and South Ethiopia (74%) (10). Studies revealed that socio-demographic factors such as residence area, nearness of health facility, and maternal education can affect the level of maternal health care utilization, particularly institutional deliveries (37, 38). Therefore, the discrepancy of these findings might be due to the socio-economic characteristics of the study population and the availability of nearby health facilities in the areas.

In addition, the study found that the institutional delivery across the 6 years appears to decrease. The conflict or any political instability could negatively affect the maternal and child health care services (39–41) and mothers receive fewer ANC check-ups during political instability which hindered them not delivering t health facility (42). Thus, the decreasing level of institutional delivery in this study could be related to the political instability that happened in our country since 2016 and there is also the effect of the COVID-19 pandemic which has been happening worldwide. COVID-19 has negatively affected maternal institutional delivery due to lockdown, restriction of movements, and closure of health facilities (43–45).

The odds of institutional delivery were increased by 54% among mothers who were unable to read and write, compared to those who were unable to read and write. This finding was comparable with the studies conducted in Sudan (46), Ghana (47, 48), India (49), and Nigeria (50). This might be because uneducated mothers may not aware of the cultural acceptability of the delivery services given at health facilities, unable to understand the message prepared and conveyed through some reading materials because of unable to read and write (46, 51, 52). Moreover, the decisive role of the mothers could affect the place of delivery. Thus, the role of mothers in deciding the place of delivery could be affected by the husband or other elder mothers, especially among mothers who had no formal education (53, 54).

Institutional delivery was 2.33 and 7.18 times more likely among mothers residing in a semi-urban area and urban area respectively, compared to those who were residing in the rural area. These findings are consistent with the multilevel analysis in Ethiopia (11), and supported by the studies from Eritrea (53), Ghana (47), Indonesia (55), Kenya (56), and Ethiopia (22). This might be because of mothers residing in urban areas are being close to health facilities, exposure to media, no transportation difficulties, and good road conditions (52). Due to relatively less empowerment of women residing in rural areas, the decision of place of delivery might be determined by the head of the household and elder mothers (57).

Mothers who received antenatal care had 73% higher odds of institutional delivery, compared to those who did not receive antenatal care. This finding is aligned with the studies from Ethiopia (36, 58–60), Pradesh, India (61), Tanzania (62), and Bangladesh (63). One of the services given by ANC includes counseling for the place of delivery preparation (64). Thus, the mothers who attend ANC follow-up may more likely to get the services given including the counseling on the delivery preparation such as labor-induced signs, early warning signs, and place of delivery.

Rich mothers had 11% higher odds of institutional delivery, compared to those who were poor. This is supported by previous studies (11, 65). This might be due to access to health care services, transportation costs, and additional costs. Hence, women who can pay for such costs are more likely to deliver at health facilities.

### Implications of the Study

This study highlights the value of using HDSS data to estimate the institutional delivery size to inform policy and build locally suitable programs. Furthermore, the study suggests that giving special emphasis to delivery care by using village health care workers with proper training, to provide emergency obstetric care in the home during political instability and the occurrence of a pandemic. These strategies suggest the role of properly designed and implemented policies, whether initiated by government agencies or NGOs, to mitigate the effect of violent conflict and the occurrence of a pandemic on maternal health care utilization.

The main limitations of this study are since the study was a secondary data analysis there were incomplete or mislabeled variables, restricted variable data, and inconsistent values. In addition, the data for important variables like frequency of ANC and distance from the nearest health facility were not collected on the HDSS.

## Conclusion

The magnitude of institutional delivery was low and has shown a decreasing trend over the observed 6-year period. The major determinants for institutional delivery were a place of residence, mother's education, wealth index, and antenatal care. Community-based interventions should be strengthened to reverse the decreasing trend of institutional delivery which is a critical and proven intervention for reducing maternal and neonatal mortality. Targeted information education and communication should be provided to uneducated mothers. Moreover, strategic actions are required to promote antenatal care and attention should be given to the communities living in the rural areas and for those who are poor.

## Data Availability Statement

The data that support the findings of this study are available by requesting Kersa HDSS, Haramaya University. The datasets analyzed during the current study are available from the corresponding author upon reasonable request.

## Ethics Statement

Kersa HDSS has obtained ethical clearance from the National Ethical Review Committee and Haramaya University College of Health and Medical Science, Institutional Health Research Ethical Review Committee (IHRERC). During the surveillance process, written consent was obtained from each participant from the head of the family or eligible adult among the family members. For participants who cannot read and write, the data collectors were read the consent, and obtained the fingerprint sign. Confidentiality of information obtained from the study participants was assured throughout the study. Personal identifiers were removed from the data. We accessed the raw data after authorization was granted from Haramaya University, Kersa HDSS office by explaining the goal of our study. All methods were performed following the relevant guidelines and regulations.

## Author Contributions

TR has conceptualized the manuscript, performed data curation, statistical analysis, interpretation, and drafting of the manuscript. GA, MY, BM, MK, BN, AB, AA, and YD have critically revised the research starting from its inception, design, and planning of the study, statistical analysis, interpreted the findings, and have participated in the drafting of the manuscript. MD has participated in data collection, data analysis, revision of the paper, and has participated in the drafting of the manuscript. All authors reviewed and approved the final version of the manuscript.

## Conflict of Interest

The authors declare that the research was conducted in the absence of any commercial or financial relationships that could be construed as a potential conflict of interest.

## Publisher's Note

All claims expressed in this article are solely those of the authors and do not necessarily represent those of their affiliated organizations, or those of the publisher, the editors and the reviewers. Any product that may be evaluated in this article, or claim that may be made by its manufacturer, is not guaranteed or endorsed by the publisher.

## Abbreviations

AOR: Adjusted Odds Ratio
CI: Confidence Interval
EDHS: Ethiopia Demographic and Health Survey
HDSS: Health and Demographic Surveillance System
MMR: Maternal Mortality Ratio
WHO: World Health Organization.
